# Dopamine dips during unrewarded actions promote punishment-resistant reward seeking

**DOI:** 10.64898/2026.08.01.742224

**Published:** 2026-08-03

**Authors:** Nkatha Mwenda, Jacob A. Nadel, Tong Shen, Jillian L. Seiler, Talia N. Lerner

## Abstract

Punishment-resistant reward-seeking, a hallmark of addiction, is less prominent in females than males. We found that chronic estradiol manipulations increased punishment resistance in female mice without increasing dopamine peaks on rewarded actions as expected, instead exaggerating dopamine dips on unrewarded actions; optogenetically mimicking these dips accelerated punishment resistance in females and males. These results suggest dopamine dips suppress learning from unrewarded actions, consistent with policy-based accounts of dopamine function.

## Full Text Availability

The license terms selected by the author(s) for this preprint version do not permit archiving in PMC. The full text is available from the preprint server.

